# Optimization of Resin Composition for Zirconia Ceramic Digital Light Processing Additive Manufacturing

**DOI:** 10.3390/polym17060797

**Published:** 2025-03-18

**Authors:** Ning Kuang, Minghui Xiao, Hao Qi, Wenjie Zhao, Junfei Wu

**Affiliations:** 1 College of Electromechanical Engineering, Qingdao University of Science and Technology, Qingdao 266061, China; 2 College of Sino-German Science and Technology, Qingdao University of Science and Technology, Qingdao 266061, China

**Keywords:** additive manufacturing, DLP, zirconia, photosensitive resin

## Abstract

In ceramic digital light processing (DLP) additive manufacturing, the photosensitive resin, which acts as a carrier for ceramic particles, must exhibit suitable curing performance, curing strength, and viscosity. This ensures both the bonding strength of the fabricated ceramic parts and the dimensional accuracy of the ceramic green body. In this study, various photosensitive resin monomers were investigated in depth to formulate resins containing monofunctional, bifunctional, and multifunctional groups. Their rheological and curing properties were analyzed theoretically and experimentally. Different resin slurry systems were prepared and printed using DLP technology, and their mechanical properties were tested and compared. The effect of photoinitiator content on the curing behavior of the resin was examined, and the optimal photoinitiator concentration was identified. Based on the optimized resin, a zirconia ceramic slurry with 56 vol% solid content was prepared. After DLP printing, debinding, and sintering, dense zirconia ceramic samples with a relatively uniform grain structure were obtained, exhibiting a bending strength of 766.85 MPa. These results significantly expand the potential applications for zirconia ceramic components with complex geometries.

## 1. Introduction

With the rapid advancement of modern industrial technologies and design concepts, the demand for zirconia ceramics with complex structures, fine features, and high precision is increasing. Due to their excellent toughness [1], strength, wear resistance [2], and good biocompatibility [3], zirconia ceramics are widely used in various industrial applications such as high-performance cutting tools and bearings as well as in medical devices, including dental restorations and artificial joints [4,5,6]. However, the high hardness, brittleness, and processing difficulties of zirconia ceramics pose significant challenges when it comes to manufacturing complex-shaped components using traditional machining methods.

While techniques such as isostatic pressing, injection molding, and gel casting can be employed to produce relatively complex-shaped ceramics [7], these methods are heavily reliant on molds and come with drawbacks, such as high mold fabrication costs, complicated processes, long production cycles, and limitations in producing parts with intricate structures. These challenges hinder the broader application of complex-shaped ceramic components.

Additive manufacturing, or 3D printing, operates by building up a model layer by layer to create the final structure. This technology offers several advantages, including fast production speeds, high forming precision, and low material costs [8,9]. According to the ISO/ASTM 52900 standard [10], additive manufacturing processes are categorized into seven distinct types based on their material forms and processing techniques. Two of these processes, namely Material Extrusion and Directed Energy Deposition, utilize wire as their primary raw material. Another three processes—Material Jetting, Binder Jetting, and Powder Bed Fusion—employ powder as their fundamental material form. The remaining process, Vat Photopolymerization, uses liquid materials such as resin and slurry as its raw material input. In recent years, additive manufacturing has found widespread use in the production of ceramic materials, including alumina [11,12,13], zirconia [14,15], silicon carbide [16,17], silicon nitride [18,19], aluminum nitride [20,21], etc.

**Figure 1:**
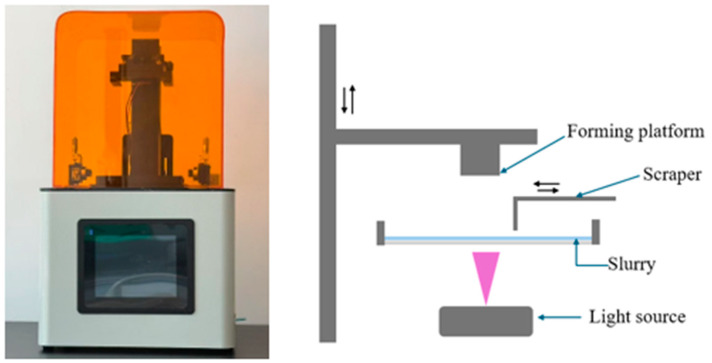
DLP printer and schematic diagram.

Ceramic additive manufacturing primarily encompasses techniques such as laminated object manufacturing (LOM), fused deposition modeling (FDM), selective laser melting (SLM), and photopolymerization [22]. Among these, photopolymerization-based methods are including stereolithography (SL), liquid crystal display (LCD), and digital light processing (DLP). The DLP technology, which is based on surface exposure, has become particularly mature [23,24,25,26]. DLP technology works by using a high-resolution digital projector to project images for layer-by-layer curing, which are then stacked to form the object. Since each layer is formed in a single exposure, DLP offers high precision, speed, and cost-efficiency [27]. DLP systems are classified into top-exposure and bottom-exposure types based on the positioning of the light source [28,29]. In this experiment, a bottom-exposure DLP printer was used, where the light source projects from beneath the resin tank, curing the resin and building the component layer by layer on the formation platform, as shown in Figure 1.

**Figure 2:**
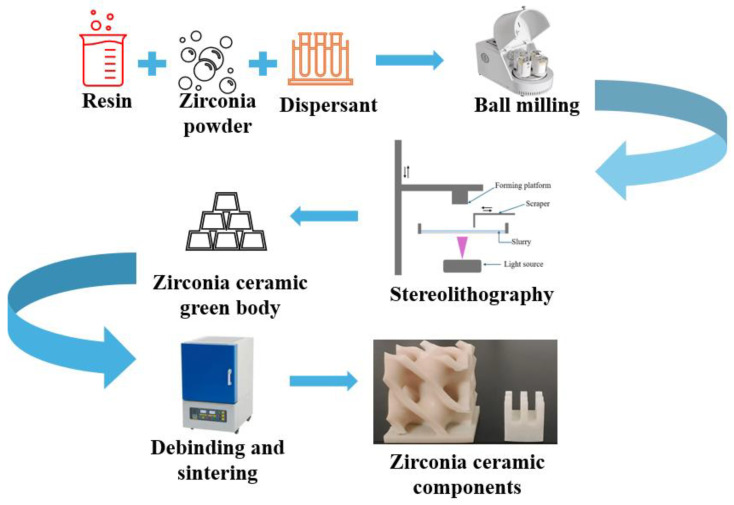
Three-dimensional printing zirconia ceramic fabrication process.

An essential step in 3D printing zirconia ceramics using DLP technology is the incorporation of zirconia powder into a pre-prepared photosensitive resin suspension, followed by the addition of dispersants and other additives to ensure the stability of the slurry, as shown in Figure 2. The preparation of the photosensitive resin involves selecting appropriate reactive diluents and adding a specific amount of photoinitiators.

Given the layer-by-layer nature of the photocuring process, it is crucial for the photosensitive resin to exhibit good fluidity and low viscosity as well as excellent photocuring performance [30]. Ceramic products printed from slurries with high ceramic solid content generally display superior properties, such as higher hardness and strength. However, a common challenge is the increase in slurry viscosity, which leads to poor fluidity and negatively impacts print quality [25]. Therefore, selecting the suitable photosensitive resin formulation is key to ensuring slurry stability while balancing the demands of high solid content and low viscosity.

Monomers are the most critical components of a photosensitive resin. Depending on the number of reactive functional groups in each molecule, monomers can be categorized as monofunctional, bifunctional, or multifunctional. Monomers not only help control the viscosity of the resin system, but also participate in the photocuring reaction, directly impacting key aspects such as the curing rate and precision. Photoinitiators, essential for initiating the polymerization of the resin monomers during the curing process [31,32], are typically present in much lower concentrations than the reactive diluents, usually between 1% and 5% and rarely exceed 10% [33]. Photoinitiators can be classified into UV or visible light types based on their energy absorption characteristics. Different photoinitiators absorb at distinct wavelengths, and the selection of the appropriate photoinitiator is important for achieving optimal curing performance [34].

The polymerization reaction initiated by the photoinitiator follows a free radical polymerization mechanism. With light exposure, the photoinitiator (PI) is excited from its ground state to an active singlet or triplet state, generating active free radicals (R) that can initiate the polymerization process. These free radicals then interact with the reactive monomer (M), abstracting electrons from the double bonds and causing the bonding electron pairs to become isolated, thus forming active monomer species.

(1) $$\mathrm{PI}\overset{\;\mathrm{hv}\;}{\to }R\cdot ,$$

(2) $$R\cdot +M\overset{\;\mathrm{hv}\;}{\to }\mathrm{RM}\cdot ,$$

The active species of the reactive monomer exhibit high chemical reactivity, capable of cleaving the π-bonds of double-bonded molecules to form new active species. These newly formed species then continue to participate in addition reactions, promoting chain growth and leading to the formation of active chains with terminal active centers, denoted as RM_n_·, as shown below:

(3) $$\mathrm{RM}\cdot +\left(n-1\right)M\overset{\;\mathrm{hv}\;}{\to }RM_{n}\cdot$$

During the addition reaction, the active chain RM_n_· may interact with another active chain, causing the active center to lose its original reactivity and resulting in the formation of a polymer.

The viscosity of monofunctional monomers and bifunctional monomers compared to multifunctional monomers is lower; they can enhance the rheological properties of the resin. Multifunctional monomers can accelerate the curing rate and form a highly crosslinked network structure, so the printed part has high mechanical performance. Due to the differences in the types of functional groups, even if the number of functional groups is the same, the monomers will have different characteristics. Therefore, taking into account the characteristics of different numbers of functional groups as well as the properties of each functional group, preparing a mixed photoinitiator resin system is one of the core contents of the research on DLP 3D printing. This in turn influences the viscosity and curing characteristics of the zirconia ceramic slurry.

Recent research has shown several advancements in resin-based ceramic suspensions. Xu et al. [35] enhanced the adhesion properties of resin-based ceramic suspensions by reducing double bond density and volumetric shrinkage. A UV-cured alumina suspension composed of 20 vol% isobutyloxyethyl alcohol (IBOA, monofunctional), 50 vol% 1,6-Hexanediol diacrylate (HDDA, bifunctional), and 30 vol% ethoxylated pentaerythritol tetraacrylate (PPTTA, multifunctional) demonstrated excellent adhesion and suitable curing performance. Dang et al. [28] substituted part of the PPTTA monomer with ethoxylated trimethylolpropane triacrylate (ETPTA, multifunctional) and introduced oligomers, reducing the critical exposure energy to 14.88 mJ/cm^2^ and increasing the conversion rate to 98.5%. Johansson et al. [36] added non-reactive components 2-[[2-(Benzoyloxy)ethyl]amino]ethanol (BEA) and polyethylene glycol (PEG-200) to the photosensitive resin, which helped reduce polymerization shrinkage and modified the thermal decomposition of the polymer matrix. This adjustment minimized layer delamination and interlayer cracks after thermal treatment, achieving a relative density close to 99%. Komissarenko et al. [37] used a mixed bifunctional monomer system and found that the formation of a robust polymer network during the curing process led to increased green body stiffness, making it easier to print high-resolution, intricate structures. Gong et al. [38] employed two diluents with significantly different molecular weights, HDDA and acryloyl morpholine (ACMO, monofunctional), expanding the temperature range for resin decomposition during ceramic body heat treatment, effectively removing 12% of the resin at lower temperatures.

From these studies, it is clear that monomers with different functional groups exhibit distinct rheological and photocuring properties, influenced by factors such as molecular weight, molecular structure, and the properties of their functional groups. In order to achieve photosensitive resins with optimal rheological and photocuring properties, it is common to use a combination of different monomers. Monofunctional monomers, which have low viscosity, improve the rheological properties of the resin and can effectively reduce curing shrinkage. Therefore, selecting the suitable monofunctional monomers and the appropriate proportions can significantly enhance the performance of zirconia ceramic slurry. However, at present, there are relatively few studies on multi-component photosensitive resin systems, which include monofunctional monomers, bifunctional monomers, and multifunctional monomers. Especially for zirconia ceramic slurry, there is a lack of systematic research on the influence of different monomers in photosensitive resin systems.

In this study, a combination of monofunctional, bifunctional, and multifunctional monomers was selected while investigating the composition of photosensitive resins. Through resin composition optimization, an appropriate resin was developed, and with this resin, the printed specimens exhibited high tensile and bending strengths. Additionally, the impact of photoinitiator content on resin performance was also evaluated, resulting in optimal photoinitiator content. The zirconia ceramic slurry prepared with this resin was subjected to printing, debinding, sintering, and testing, resulting in significant improvements in properties of the ceramic finish part.

## 2. Materials and Methods

### 2.1. Materials

In this study, zirconia powder (median particle size D50 = 1 µm, Shandong Sitaili Metal Materials Co., Ltd., Jinan, China) was used, with yttria (Shanghai Liantian Materials Technology Co., Ltd., Shanghai, China) serving as a sintering additive. The photoinitiator used was phenylbis (2,4,6-trimethylbenzoyl) phosphine oxide (819, Shanghai Guangyi Chemical Co., Ltd., Shanghai, China), while BYK110 (Dongguan Haoyouduo New Materials Co., Ltd., Dongguan, China) was used as a dispersant. The photosensitive resin monomers used in the experiment are listed in Table 1.

### 2.2. Preparation and Characterization of Photosensitive Resin

The viscosities of the selected monomers were measured using a rotational viscometer. Monofunctional monomers ACMO and HEMA were chosen and mixed with bifunctional monomer HDDA and multifunctional monomer TMPTA in order to distribute the experimental points uniformly, which can cover more combinations of factors and levels. To save time and cost, comprehensive experimental results can be obtained by reducing the number of experiments. Based on the concept of uniform design [39], eight formulations were designed, as shown in Table 2, and three-component photosensitive resins were prepared, with 5% of photoinitiator added to each formulation. The mixtures were stirred for 60 min using a magnetic stirrer at a speed of 500 rpm (Table 3).

**Figure 3:**
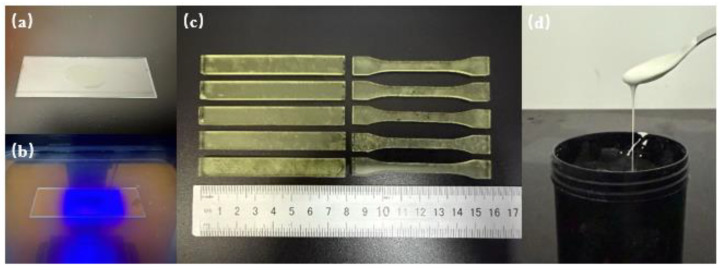
Cure depth test: (**a**) before curing, (**b**) while curing; (**c**) printed bending specimens and tensile specimens; (**d**) zirconia slurry.

A total of 16 groups resin from the two prepared formulations were tested for curing depth. The resin was evenly spread on a glass slide, as shown in Figure 3a. Under a light intensity of 3100 μW/cm^2^ and an exposure time of 5000 ms, the glass slide was irradiated using the 3D printer, as shown in Figure 3b. After curing, the uncured resin was wiped off, and the cured layer together with glass slide was measured by micrometer. After subtracting the thickness of the slide, the thickness of the cured layer can be calculated. To ensure repeatability, five samples of each resin and 3 points of each sample were tested.

The two resin formulations (16 groups in total) were printed using a DLP resin printer. The dimension of printed bending test specimens was 80 × 10 × 4 mm and that of the tensile test specimens was 75 × 10 × 3 mm, as shown in Figure 3c; the layer thickness of both specimens was 50 μm. The printing light intensity was 8530 μW/cm^2^, with an exposure time of 3000 ms. A testing machine was used to perform three-point bending strength tests on the bending test specimens at a loading rate of 1 mm/min and tensile strength tests on the tensile test specimens at the same loading rate. Each resin was tested on 5 printed samples to ensure repeatability of the measurements. The testing methods and requirements are in accordance with standards ISO 178:2001 [40] and ISO 527:2012 [41].

Following the comparative testing, the three-component photosensitive resin made up of HDDA, TMPTA, and HEMA was selected for its superior performance. The monomer content was based on resin formulation #8 from Table 2, with photoinitiator added in amounts ranging from 1 wt% to 5 wt%. Under the same conditions as before, curing depth as well as bending and tensile test specimen underwent printing and performance testing.

### 2.3. Preparation of Zirconia Ceramic Slurry

HDDA, TMPTA, and HEMA were selected as the photosensitive resin monomers to prepare a zirconia ceramic slurry with 56 vol% solid content. As tested, when solid loading was higher, the viscosity was so high that it was difficult to print. A specified amount of zirconia ceramic powder and a small quantity (5 wt% of zirconia powder) of sintering additive were added to the prepared photosensitive resin to form a ceramic premix. To enhance the slurry’s stability, a dispersant (3 wt% of zirconia powder) was incorporated. The slurry was then ball-milled using a planetary ball mill at 350 rpm, with alternating forward and reverse rotations for 10 h each, resulting in a well-dispersed zirconia ceramic slurry, as shown in Figure 3d.

### 2.4. Ceramic Sample Fabrication

Zirconia ceramic green bodies were printed using a DLP ceramic printer. According to the layer thickness of 3D model and in order to decrease the printing time, we increased the light intensity and reduced exposure time. The printing light intensity was 12,330 μW/cm^2^, with an exposure time of 6500 ms, and the layer thickness was 50 μm. The printed green bodies had dimensions of 35 × 4 × 3 mm. To determine the temperature at which organic compounds decompose, a thermogravimetric analysis was performed. The zirconia green body was heated from room temperature to 700 °C at a rate of 5 °C/min. Based on the TG-DTG results, appropriate debinding and sintering programs were established. Zirconia green body was debound at 600 °C in air atmosphere in debinding furnace for 2 h and then sintered in the sintering furnace with a heating rate of 5 °C/min to 1600 °C for 2 h.

### 2.5. Mechanical Properties

**Figure 4:**
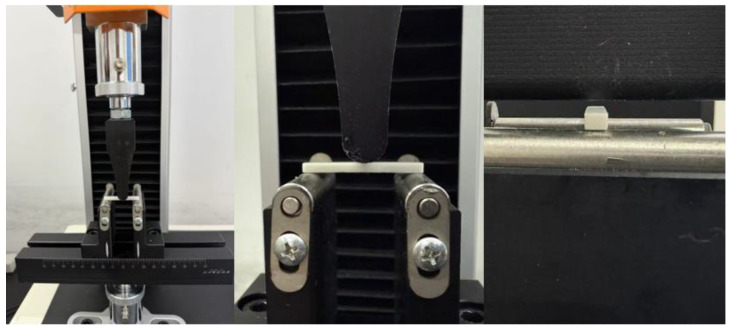
Three-point bending test.

The sintered ceramic specimens were tested for three-point bending strength using a digital electronic universal testing machine, as shown in Figure 4, at a testing rate of 0.5 mm/min. To ensure repeatability, five samples were tested. The testing methods and requirements were in accordance with standard ISO 14704:2000 [42].

## 3. Results and Discussion

### 3.1. Rheological Properties of Photosensitive Resin

**Figure 5:**
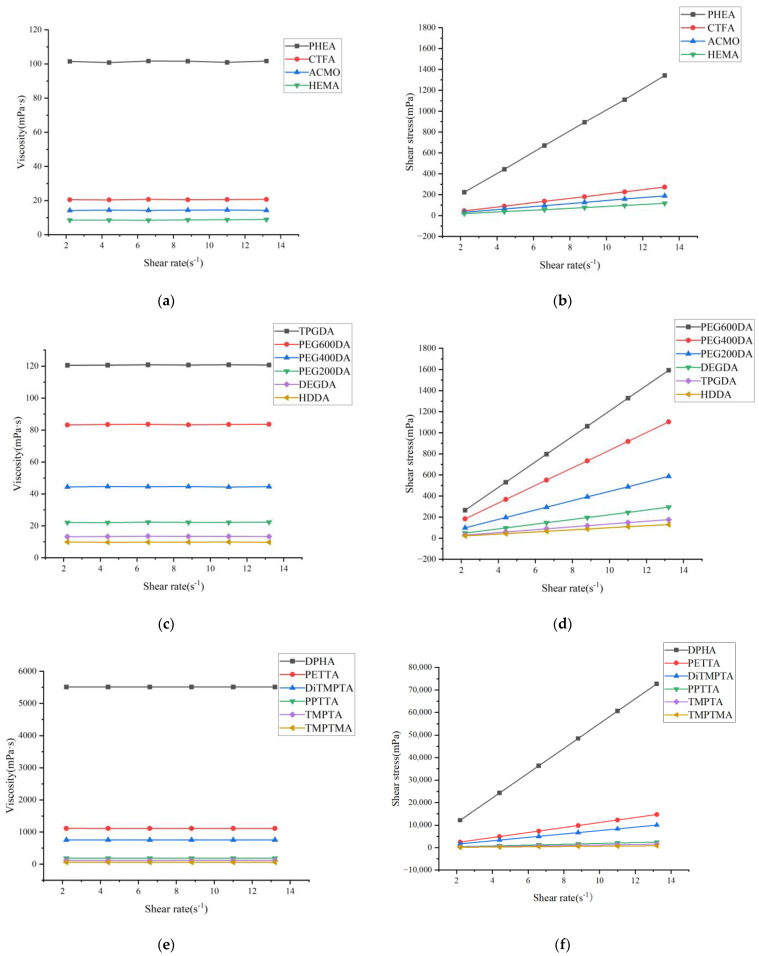
Relationship between shear viscosity, shear stress, and shear rate of different monomers: (**a**,**b**) monofunctional monomers; (**c**,**d**) bifunctional monomers; (**e**,**f**) multifunctional monomer.

The rheological analysis of monomers with varying numbers of functional groups revealed that viscosity increases as the number of functional groups increases, as shown in Figure 5a–f.

The viscosity and shear stress of multifunctional monomers are hundreds of times higher than monofunctional and bifunctional monomers. This is because more functional groups lead to higher molecular weight and more complex structures, which in turn result in more complete crosslinking during the photocuring process [43]. Even among monomers with the same number of functional groups, viscosity can vary due to differences in the properties of these groups, with more complex structures leading to higher viscosity.

In this study, ACMO and HEMA, both low-viscosity monofunctional monomers, were selected for experimentation. HDDA was chosen as the bifunctional monomer due to its lowest viscosity. In multifunctional monomers, TMPTMA and TMPTA have the lowest viscosity based on our previous experience of printing, TMPTA has better print stability; therefore, TMPTA was selected as the multifunctional monomer. These four monomers were used to prepare multi-component photosensitive resins.

**Figure 6:**
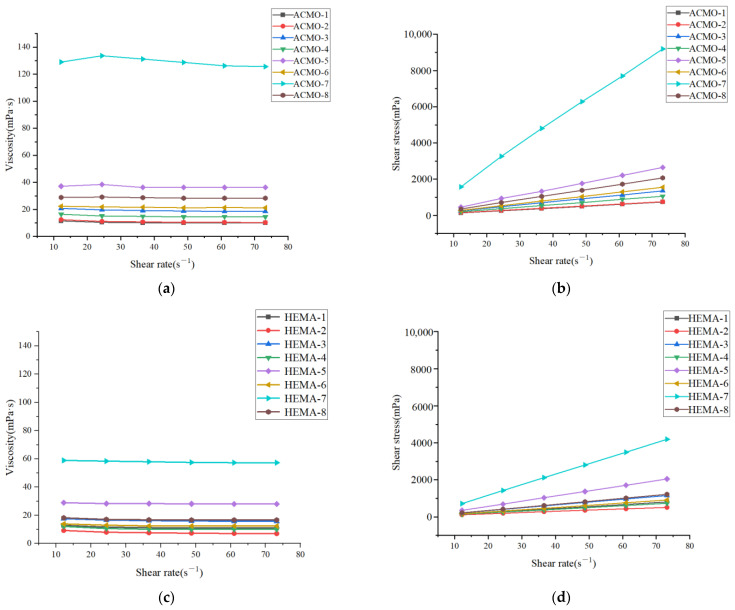
Relationship between shear viscosity, shear stress, and shear rate of different resins: (**a**,**b**) formula 1; (**c**,**d**) formula 2.

As the monomers contain different active groups, the photocuring reactivity and intensity of the monofunctional monomers ACMO and HEMA are also different. The study followed a formulation design approach, conducting preparation experiments and printing with two distinct formulations: formula 1, which used HDDA, TMPTA, and ACMO, and formula 2, which used HDDA, TMPTA, and HEMA. As shown in Figure 6, the viscosity of the mixed resins remained constant with varying shear rates, and the shear rate-shear stress curves were straight lines passing through the origin, indicating that the resins behaved as Newtonian fluids. For Newtonian fluids, viscosity is mainly influenced by temperature and material composition, which means the rheological properties of the resin are closely tied to its composition.

Viscosity measurements of formula 1 and formula 2 showed that viscosity was directly proportional to the content of multifunctional monomers in the formulation. Resin #7, which had the highest content of multifunctional monomers, exhibited the highest viscosity, while resin #2, with the least content of multifunctional monomers, had the lowest viscosity. This is because multi-functional monomers have much higher viscosity compared to monofunctional and bifunctional monomers and thus have a decisive effect on the overall viscosity of the mixed resin system. Moreover, when comparing the viscosities of formulas 1 and 2 with consistent content of monofunctional, bifunctional, and multifunctional monomers, formula 2 exhibited lower viscosity than formula 1. Based on the rheological performance, formula 2, which included HDDA, TMPTA, and HEMA, performed better and was therefore selected for further analysis. The regression equation between viscosity Y_1_ and components X_i_ (as in Table 2, i = 1, 2, 3) was derived through regression equation.

Y_1_ = 101.39 − 112.76 X_1_ − 91.09X_3_, (4)

The regression equation’s correlation coefficient is R^2^ = 0.991, indicating that the equation accurately reflects the relationship between resin viscosity Y_1_ and components X_i_ (i = 1, 2, 3). The *p*-values of X_i_ (i = 1, 2, 3) are 0.039, 0.119, and 0.041, respectively; the significant level α is 0.05; and the X_2_ regression is not significant. In the regression equation, it can be concluded that the viscosity of the resin is primarily related to the monomers, specifically HDDA and HEMA. The absolute value of the coefficient for the variable related to HDDA is 112.76, slightly higher than the absolute value of 91.09 for the variable related to HEMA. This suggests that the content of the monomers has the most significant impact on the viscosity reduction of the resin mixture, with the content of HEMA also playing a considerable role. The reason for this is that monofunctional and bifunctional monomers have the advantages of low viscosity and strong dilution capability.

### 3.2. Photocuring Performance of Photosensitive Resin

**Figure 7:**
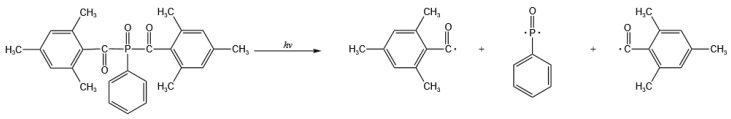
819 photodecomposition mechanism.

During light exposure, the photoinitiator in the photosensitive resin system generates free radicals that can initiate the polymerization of monomers. This leads to the polymerization and crosslinking of active monomers within the photosensitive materials, ultimately forming a three-dimensional network structure. In this study, the 3D printer used has a light source with a wavelength of 405 nm, and therefore, photoinitiator 819 was selected. The photodecomposition products of 819 consist of two Trimethylbenzoyl radicals and one Phenylphosphine oxide radical, as shown in Figure 7, all of which are highly reactive free radicals that effectively trigger the polymerization process.

The relationship between irradiation light intensity and cure depth can be explained by Beer-Lambert’s semi-logarithmic mode, illustrated by Equation (5) [44], which means that the light energy decays exponentially along the direction of the irradiation depth.

(5) $$C_{d}=D_{p}\ln\left(\frac{E}{E_{c}}\right),$$

Here, the curing depth C_d_ (μm) is determined by the projection depth D_p_ (μm), which serves as a sensitivity parameter indicating the depth at which the beam intensity decreases to 1/e^2^ of its surface value at the resin interface, and the critical exposure energy E_c_ (mJ·cm^−2^), which represents the minimum energy required to initiate monomer polymerization. Both D_p_ and E_c_ are intrinsic material properties of the resin independent of light exposure conditions. The parameter E denotes the actual exposure energy (mJ·cm^−2^) delivered during the printing process, which is depends on printing light intensity and exposure time.

**Figure 8:**
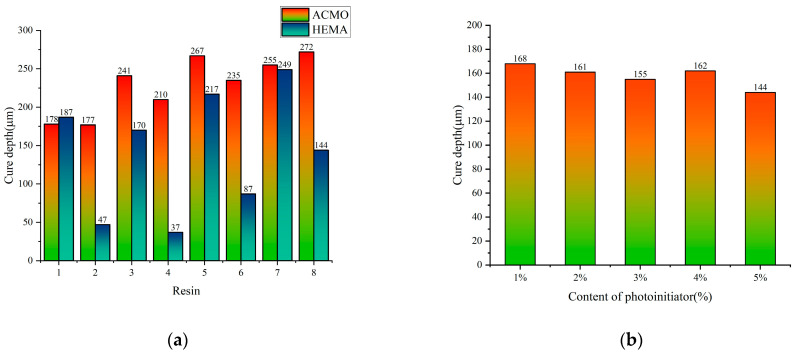
(**a**) Cure depth of formula 1 and formula 2; (**b**) relationship between cure depth and different content photoinitiators.

In order to compare the photosensitivity of each formulation, the cure depth test has been determined, and the results of the average cure depth are shown in Figure 8a; the cure depth for formula 1, which uses ACMO, is higher, while in formula 2, which uses HEMA, the cure depths for resin #2, #4, and #6 are smaller. This suggests that when the content of TMPTA is lower and the content of HEMA is higher, the reactivity of the photosensitive resin decreases. The interaction between monofunctional and multifunctional monomers plays a crucial role in the crosslinking polymerization process.

**Figure 9:**
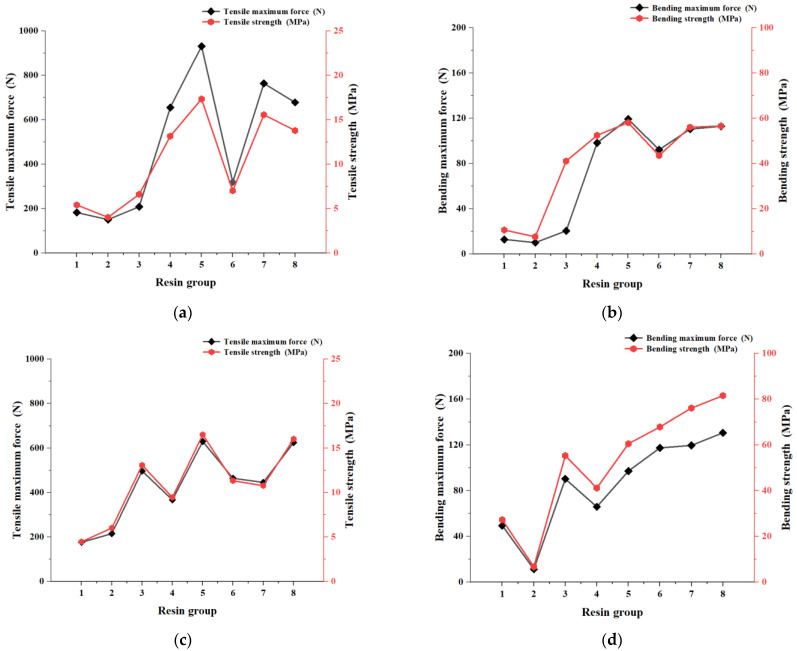
(**a**) Tensile maximum force and tensile strength of formula 1; (**b**) bending maximum force and bending strength of formula 1; (**c**) tensile maximum force and tensile strength of formula 2; (**d**) bending maximum force and bending strength of formula 2.

The average value of the mechanical properties of the printed specimens is shown in Figure 9. With the same printing parameters, the samples of resin #5 of formula 1 and also #5 from formula 2 exhibited the highest tensile strength within their respective formulas. Compared to formula 2, resin sample #5 of formula 1 showed a 4.75% higher tensile strength. For bending strength, resin #8 from formula 2 and resin #5 from formula 1 displayed the highest values in their respective formulas, resin #8 from formula 2 achieving a 28.75% higher bending strength than resin #5 from formula 1. Taking the bending strength of formula 2 as an example, the regression equation between the resin’s bending strength Y_2_ and its components X_i_ (as in Table 2, i = 1, 2, 3) was derived through the following regression equation:

Y_2_ = 21.21 − 3.56X_1_ − 3.66X_2_ − 3.64X_3_, (6)

The correlation coefficient R^2^ = 0.9011 of this regression equation indicates that the equation accurately captures the relationship between the resin’s bending strength Y_2_ and its components X_i_ (i = 1, 2, 3). The *p*-values of X_i_ (i = 1, 2, 3) are 0.056, 0.041, and 0.043, respectively, and the significance level α is 0.05. According to the equation, the absolute values of the coefficients for the independent variables are 3.56, 3.66, and 3.64, respectively. The coefficients of the independent variables X_2_ and X_3_ are all larger than those for the variable X_1_, suggesting that the bending strength of the resin is more strongly influenced by the combined effect of the components. Specifically, the content of TMPTA and HEMA monomers significantly impacts the bending strength, with the TMPTA monomer having the biggest effect on the bending strength of the photosensitive resin.

Considering the overall impact on the final product’s performance, resin #8 of formula 2 demonstrates superior performance. As a result, this resin was selected for further testing.

**Figure 10:**
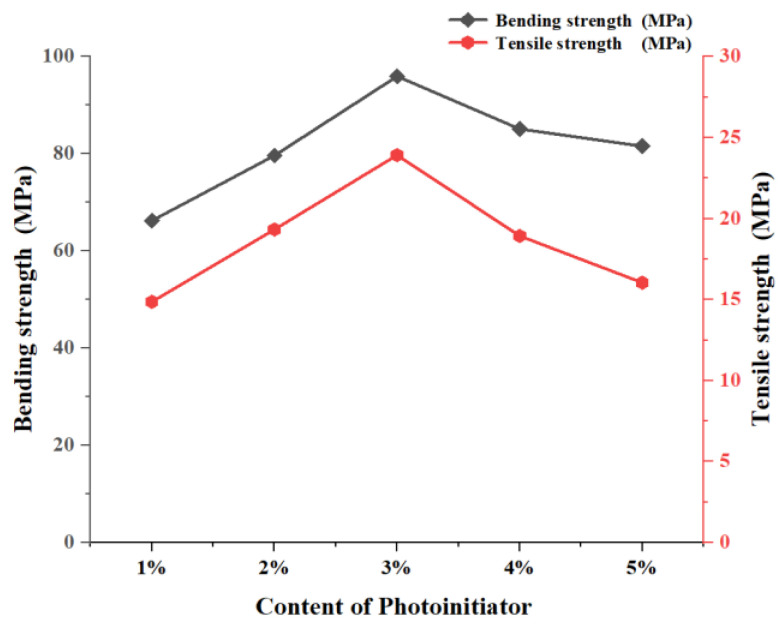
Relationship between photoinitiator content and specimen tensile/bending strength.

As shown in Figure 8b, the average cure depth of resin #8 of formula 2 remains nearly constant with the addition of 1 wt% to 5 wt% photoinitiators, indicating that the photoinitiator content has minimal impact on the curing depth when the monomer composition and content are constant. Figure 10 illustrates the effect of adding 1 wt% to 5 wt% photoinitiators to resin #8 of formula 2 on the average tensile and bending properties of the printed parts. In this experiment, the best mechanical performance was achieved when photoinitiator 819 was added at a concentration of 3 wt%. However, adding more than a 3 wt% photoinitiator led to a decline in the mechanical properties of the printed specimens. This decline may be due to an excess of the photoinitiator, which could result in some of it failing to interact with the active monomers, leading to side reactions that negatively impact the printing performance.

### 3.3. TG-DTG Analysis

**Figure 11:**
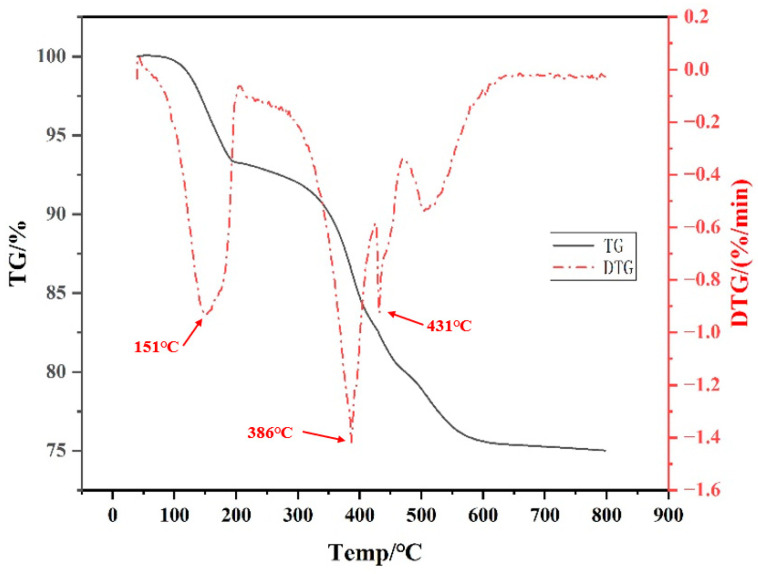
TG-DTG curve of printed zirconia ceramic green body.

As shown in the TG-DTG results in Figure 11, the decomposition temperatures of the organic materials are 151 °C, 386 °C, and 431 °C, respectively. By 600 °C, the thermal decomposition is nearly complete, and the mass of the green body stabilizes. Therefore, during the debinding process, a slow heating rate is maintained up to 100 °C to ensure complete moisture removal without causing cracking. Additionally, a holding time is applied around the three identified temperature peaks.

### 3.4. Properties of Zirconia Ceramics

**Figure 12:**
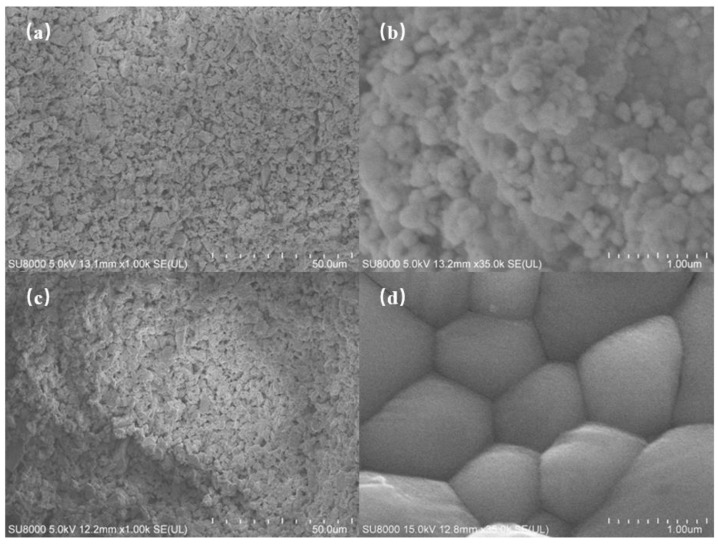
SEM images of microstructure: (**a**,**b**) before sintering; (**c**,**d**) after sintering.

The SEM micrographs of the ceramic samples before and after sintering are shown in Figure 12. Before sintering, the ceramic’s internal structure was loose, with small and uneven grain sizes. After sintering, the structure became more compact, though some porosity remained. Small pores may have formed during the debinding and sintering processes. The grain size of the sintered ceramic showed a noticeable increase, becoming more uniform, indicating that the chosen sintering temperature and time were appropriate.

Given the widespread application of zirconia ceramics in dental prosthetics and structural components, bending strength serves as a critical indicator of material performance. This parameter is particularly valuable, as it enables the detection of internal material defects, including voids and cracks, thereby providing an effective assessment of the material’s uniformity and density. Furthermore, bending strength exhibits strong correlations with both hardness and fracture toughness, making it a reliable indirect measurement of the material’s comprehensive mechanical properties. Therefore, in this study, the bending strength is used to evaluate the mechanical properties of sintered zirconia ceramics.

**Figure 13:**
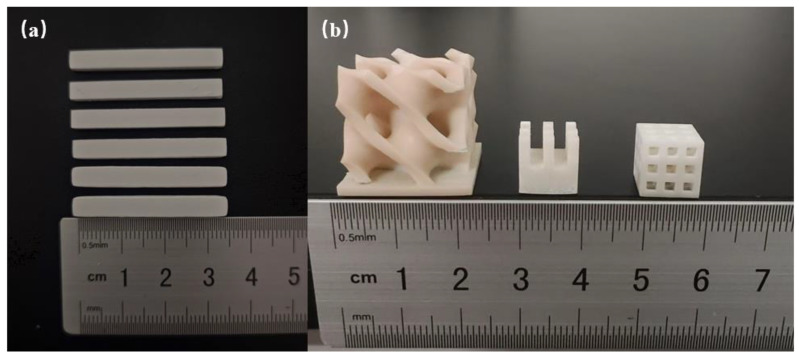
(**a**) Zirconia ceramic samples; (**b**) zirconia ceramic products manufactured by DLP.

The sintered zirconia ceramic samples, shown in Figure 13a, underwent three-point bending tests, with the measured average value of the bending strength reaching 766.85 MPa. Figure 13b presents small, dense zirconia ceramic components printed and sintered, which used the same slurry, exhibiting a smooth surface and intact structure. No delamination, intra-layer cracks, or other macroscopic defects were observed on the ceramic parts. The heat sink (middle of Figure 13b) could also be produced by injection molding or other traditional manufacturing processes, but expensive molds are needed for prototyping or small-batch production, the price is quite high, and lead time is much longer than additive manufacturing. Other more complex components such as the zirconia ceramic of triply periodic minimal surfaces (TPMS, left of Figure 13b) currently can only be accomplished through additive manufacturing.

These results further demonstrate that the resin combination explored in this study is suitable for DLP additive manufacturing of zirconia ceramics. The process can also be used to fabricate ceramic components with complex geometries, greatly expanding the potential applications for future ceramic parts, which not only encompasses zirconia. This study also has reference significance for other ceramic materials in DLP additive manufacturing, such as alumina, aluminum nitride, and silicon carbide.

## 4. Conclusions

In this study, the effects of different monomer combinations with varying numbers of functional groups on the viscosity and curing behavior of photosensitive resin systems were systematically investigated along with the influence of the photoinitiator ratio on the curing performance.

1. Monomer impact: Two distinct monofunctional monomers, ACMO and HEMA, exhibit different effects on the photocuring behavior of photosensitive resin systems. Notably, after curing, the tensile strength of the resin with ACMO is higher than that of the resin with HEMA, while the bending strength of the resin with HEMA is significantly superior to that of the resin with ACMO.
2. Photoinitiator impact: The content of the photoinitiator significantly influences the photocuring behavior. Initially, increasing the photoinitiator content led to a marked improvement in both tensile and bending strengths of the cured specimens. However, when the photoinitiator concentration exceeded 3%, further increases caused a decline in both tensile and bending strength.
3. Ceramic slurry performance: A ceramic slurry with 56 vol% solid content was prepared using the photosensitive resin containing HEMA. After printing, debinding, and sintering, the bending strength of the resulting zirconia ceramic samples reached 766.85 MPa.

This study highlights the importance of monomer selection and photoinitiator content in optimizing the properties of photosensitive resins for 3D printing of high-performance ceramic components. For further research, a wider range of monomers and photoinitiators will be covered. Other additives will also be introduced to study their effects.

## Figures and Tables

**Table 1:** Monomers selected for the experiment.

Monomer Name	Abbreviation	Number of Functional Groups	Source
Acryloyl morpholine	ACMO	1	Shanghai Guangyi Chemical Co., Ltd., Shanghai, China
Hydroxyethyl methacrylate	HEMA	1	Shanghai Guangyi Chemical Co., Ltd., Shanghai, China
Cyclic trimethylpropane formal acrylate	CTFA	1	Shanghai Guangyi Chemical Co., Ltd., Shanghai, China
2-Phenoxyethyl acrylate	PHEA	1	Shanghai Guangyi Chemical Co., Ltd., Shanghai, China
1,6-Hexanediol diacrylate	HDDA	2	Shanghai Guangyi Chemical Co., Ltd., Shanghai, China
Polyethylene glycol (200) diacrylate	PEG200DA	2	Guangzhou Lihou Trading Co., Ltd., Guangzhou, China
Polyethylene glycol (400) diacrylate	PEG400DA	2	Guangzhou Lihou Trading Co., Ltd., Guangzhou, China
Polyethylene glycol (600) diacrylate	PEG600DA	2	Guangzhou Lihou Trading Co., Ltd., Guangzhou, China
Tripropylene glycol diacrylate	TPGDA	2	Guangzhou Lihou Trading Co., Ltd., Guangzhou, China
Diethylene glycol diacrylate	DEGDA	2	Guangzhou Lihou Trading Co., Ltd., Guangzhou, China
Trimethylolpropane triacrylate	TMPTA	3	Shanghai Guangyi Chemical Co., Ltd., Shanghai, China
Di(trimethylolpropane) tetraacrylate	DiTMPTA	3	Shanghai Guangyi Chemical Co., Ltd., Shanghai, China
Ethoxylated pentaerythritol tetraacrylate	PPTTA	4	Shanghai Guangyi Chemical Co., Ltd., Shanghai, China
Pentaerythritol tetraacrylate	PETTA	4	Guangzhou Lihou Trading Co., Ltd., Guangzhou, China
Dipentaerythritol hexaacrylate	DPHA	6	Guangzhou Lihou Trading Co., Ltd., Guangzhou, China

**Table 2:** Composition of resin.

No.	X_1_ HDDA (wt%)	X_2_ TMPTA (wt%)	X_3_ ACMO/HEMA (wt%)
1	75.0	14.1	10.9
2	56.7	2.7	40.6
3	44.1	38.4	17.5
4	33.9	12.4	53.7
5	25.0	60.9	14.1
6	17.1	25.9	57.0
7	9.9	84.5	5.6
8	3.2	42.4	54.5

**Table 3:** Corresponding equipment involved in this study.

Name	Model/Grade	Specification	Manufacturer
Rotary Viscometer	NDJ-8Spro	Measuring range: 1 mPa·s—100,000 mPa·s	Shanghai Xiniulab Instruments Co., Ltd., Shanghai, China
Magnetic Stirrer	RCT-Basic	Rotational speed: 50–1500 rpm	IKA-Werke GmbH & Co. KG, Staufen im Breisgau, Germany
Planetary Ball Mill	BQM-1L	Rotational speed: 5–450 r/min	Changsha Miqi Instruments & Equipment Co., Ltd., Changsha, China
Micrometer	Q2LF0025	Precision: 0.001 mm	Deqing Shengtaixin Electronic Technology Co., Ltd., Deqing, China
UV Radiometer	LS125	Range: 0–20,000 mW/cm^2^	Shenzhen Linshang Technology Co., Ltd., Shenzhen, China
Sintering Furnace	FMJ-05/17	Maximum temperature: 1700 °C	Hefei Facerom Intelligent Equipment Co., Ltd., Hefei, China
Debinding Furnace	FMJ-07/11	Maximum temperature: 1100 °C	Hefei Facerom Intelligent Equipment Co., Ltd., Hefei, China
DLP Resin Printer	BLD-50-C1	Printing precision: 50 μm	Qingdao Breuck 3D Additive Manufacturing Co., Ltd., Qingdao, China
DLP Ceramic Printer	BLD-25-C1	Printing precision: 25 μm	Qingdao Breuck 3D Additive Manufacturing Co., Ltd., Qingdao, China
Digital Universal Testing Machine	WH-70	Maximum Load: 5000 N	Ningbo Weiheng Testing Instruments Co., Ltd., Ningbo, China
Thermogravimetric Analyzer	TG209F3	Heating and cooling rate: 0.001–200 K/min	Netzsch Instruments GmbH, Weimer, Germany
SEM Scanning Electron Microscope	SU8000	Observation magnification: 30–500 K	Hitachi High-Technologies Co., Ltd., Tokyo, Japan

## Data Availability

Data presented in this study are available on request from the corresponding author.
